# Effectiveness of janus kinase inhibitors in relapsing giant cell arteritis in real-world clinical practice and review of the literature

**DOI:** 10.1186/s13075-024-03314-9

**Published:** 2024-06-05

**Authors:** Javier Loricera, Toluwalase Tofade, Diana Prieto-Peña, Susana Romero-Yuste, Eugenio de Miguel, Anne Riveros-Frutos, Iván Ferraz-Amaro, Eztizen Labrador, Olga Maiz, Elena Becerra, Javier Narváez, Eva Galíndez-Agirregoikoa, Ismael González-Fernández, Ana Urruticoechea-Arana, Ángel Ramos-Calvo, Fernando López-Gutiérrez, Santos Castañeda, Sebastian Unizony, Ricardo Blanco

**Affiliations:** 1 Department of Rheumatology, IDIVAL, Immunopathology Group, Hospital Universitario Marqués de Valdecilla, Avda. Valdecilla s/n, Santander, ES- 39008 Spain; 2 Neurology Department, Massachusetts General Hospital, Boston, MA USA; 3 Department of Rheumatology, Complejo Hospitalario Universitario de Pontevedra, Pontevedra, Spain; 4 Department of Rheumatology, Hospital Universitario La Paz, Madrid, Spain; 5 Department of Rheumatology, Hospital Universitario Germans Trias i Pujol, Badalona, Spain; 6 Department of Rheumatology, Complejo Hospitalario Universitario de Canarias, Santa Cruz de Tenerife, Spain; 7 Department of Rheumatology, Hospital San Pedro, Logroño, Spain; 8 Department of Rheumatology, Hospital Universitario de Donosti, San Sebastián, Spain; 9 Department of Rheumatology, Hospital Universitario de Elda, Alicante, Spain; 10 Department of Rheumatology, Hospital de Bellvitge, Barcelona, Spain; 11 Department of Rheumatology, Hospital Universitario de Basurto, Bilbao, Spain; 12 Department of Rheumatology, Complejo Asistencial Universitario de León, León, Spain; 13 Department of Rheumatology, Hospital Universitario Son Espases, Palma de Mallorca, Spain; 14 Department of Rheumatology, Complejo Hospitalario de Soria, Soria, Spain; 15 Department of Rheumatology, Hospital Universitario La Princesa, IIS-Princesa, Madrid, Spain; 16 Vasculitis and Glomerulonephritis Center, Rheumatology, Immunology and Allergy Division, Massachusetts General Hospital, Boston, MA 02114 USA

**Keywords:** Giant cell arteritis, Large vessel vasculitis, Janus kinase inhibitors, Baricitinib, Tofacitinib, Upadacitinib

## Abstract

**Background:**

A substantial proportion of patients with giant cell arteritis (GCA) relapse despite standard therapy with glucocorticoids, methotrexate and tocilizumab. The Janus kinase/signal transducer and activator of transcription (JAK/STAT) signalling pathway is involved in the pathogenesis of GCA and JAK inhibitors (JAKi) could be a therapeutic alternative. We evaluated the effectiveness of JAKi in relapsing GCA patients in a real-world setting and reviewed available literature.

**Methods:**

Retrospective analysis of GCA patients treated with JAKi for relapsing disease at thirteen centers in Spain and one center in United States (01/2017-12/2022). Outcomes assessed included clinical remission, complete remission and safety. Clinical remission was defined as the absence of GCA signs and symptoms regardless of the erythrocyte sedimentation rate (ESR) and C-reactive protein (CRP) values. Complete remission was defined as the absence of GCA signs and symptoms along with normal ESR and CRP values. A systematic literature search for other JAKi-treated GCA cases was conducted.

**Results:**

Thirty-five patients (86% females, mean age 72.3) with relapsing GCA received JAKi therapy (baricitinib, *n* = 15; tofacitinib, *n* = 10; upadacitinib, *n* = 10). Before JAKi therapy, 22 (63%) patients had received conventional synthetic immunosuppressants (e.g., methotrexate), and 30 (86%) biologics (e.g., tocilizumab). After a median (IQR) follow-up of 11 (6-15.5) months, 20 (57%) patients achieved and maintained clinical remission, 16 (46%) patients achieved and maintained complete remission, and 15 (43%) patients discontinued the initial JAKi due to relapse (*n* = 11 [31%]) or serious adverse events (*n* = 4 [11%]). A literature search identified another 36 JAKi-treated GCA cases with clinical improvement reported for the majority of them.

**Conclusions:**

This real-world analysis and literature review suggest that JAKi could be effective in GCA, including in patients failing established glucocorticoid-sparing therapies such as tocilizumab and methotrexate. A phase III randomized controlled trial of upadacitinib is currently ongoing (ClinicalTrials.gov ID NCT03725202).

**Supplementary Information:**

The online version contains supplementary material available at 10.1186/s13075-024-03314-9.

## Introduction

Giant cell arteritis (GCA) is an inflammatory disorder of large and medium-sized arteries affecting people over 50 years [1, 2]. It is the most common type of vasculitis in adults in Europe and North America. The disease is characterized by the granulomatous involvement of the aorta and its main branches with complications including blindness and thoracic aortic aneurysm [1, 2].

Glucocorticoids have been the cornerstone of GCA treatment for decades at the expense of significant treatment-related toxicity and high relapse rates upon dose reduction or drug discontinuation. Other therapies, such as methotrexate, leflunomide, azathioprine, hydroxychloroquine, cyclophosphamide or tumor necrosis factor (TNF)-$a$ inhibitors have proven to be ineffective or shown mixed results [3, 4]. To date, tocilizumab, a monoclonal antibody against the IL-6 receptor (IL-6R), is the only medication with demonstrated efficacy in terms of remission maintenance and glucocorticoid-sparing [5, 6]. However, up to 40% of patients receiving tocilizumab fail treatment due to disease relapse or tocilizumab-related side effects [6, 7]. In addition, more than half of the patients responding to tocilizumab relapse upon drug discontinuation [6, 8–10]. Therefore, other treatment options are greatly needed for patients with GCA.

Advances in the understanding of the pathophysiology of GCA have paved the way for several therapies that are currently under investigation [11–13]. In the last years, the critical role of the janus kinase/signal transducers and activators of transcription (JAK/STAT) pathways in immune-mediated diseases has been therapeutically exploited with the development of JAK inhibitors (JAKi), small molecules that block the action of type I/II cytokines [14]. In GCA, CD4^+^ T-cells and macrophages, which respond to certain key mediators through the JAK/STAT system (e.g., IL-6/STAT3, granulocyte-macrophage colony-stimulating factor [GM-CSF]/STAT5, interferon [IFN]-γ/STAT1), are present in the arterial inflammatory lesions [14]. Thus, the inhibition of JAK signalling could be an effective treatment as suggested in animal models [14]. Nevertheless, the published literature on the utility of JAKi in GCA is scarce consisting in retrospective case reports and small series of patients, and a single prospective pilot study of 15 patients [15–20]. Therefore, additional data on this topic would be valuable.

We retrospectively assessed the outcomes of a series of patients with relapsing GCA treated with JAKi in a real-world setting. We also systematically searched the literature for other patients treated with JAKi and compared the group of patients in our series receiving baricitinib with patients that received this medication in the setting of the pilot study mentioned above [15].

## Methods

### Study design and patient population

We conducted an observational, retrospective analysis of patients with GCA treated with JAKi in thirteen centers in Spain and one center in United States. All patients met the 1990 American College of Rheumatology classification criteria for GCA [21], and/or had positive temporal artery biopsy or evidence suggesting vasculitis by imaging. The types of vascular imaging studies considered for the purpose of GCA diagnosis were ultrasound of the temporal arteries (i.e., halo sign), and computed tomography angiography (CTA) (i.e., diffuse, concentric mural thickening), magnetic resonance imaging/angiography (MRI/MRA) (i.e., diffuse, concentric mural thickening with or without T2 hyperintensity and/or contrast uptake), and positron emission tomography (PET) (e.g., diffuse, concentric F18 fluorodeoxyglucose [FDG] uptake) of the large arteries (e.g., aorta and main aortic branches).

Patients received JAKi at the discretion of the treating rheumatologist for disease relapsed despite the use of glucocorticoids (e.g., prednisone or methylprednisolone) and other immunosuppressants including conventional synthetic (e.g., methotrexate) and biologic (e.g., tocilizumab) immunosuppressants. A washout period corresponding to a half-life of elimination of the biologic agent was carried out between the end of biologic therapy and the start of JAKi therapy. Because this was a retrospective study of real-world data generated by a group of independent rheumatologists, there was no pre-determined criteria to select the type or dose of JAKi or the glucocorticoid tapering regimen following JAKi therapy initiation. Factors influencing providers when making those decisions may have included patient’s preference, provider’s experience and judgement, insurance authorization, cost, and safety.

### Study assessments and outcomes

Effectiveness and safety outcomes were evaluated during JAKi treatment by systematically reviewing all rheumatology notes, laboratory values and vascular imaging results available in each patient’s medical record. During follow-up, patients were seen by the rheumatology providers at variable intervals, but mostly every one to six months. Data were extracted from the medical records following a specifically designed protocol. To minimize entry mistakes, all data were double-checked.

The primary outcome was clinical remission defined as the absence of signs and symptoms attributable to GCA (e.g., headaches, jaw claudication, polymyalgia rheumatica symptoms [PMR], etc.) regardless of the value of the erythrocyte sedimentation rate (ESR) and C-reactive protein (CRP). Secondary outcomes included complete remission defined, as per the *European Alliance of Associations for Rheumatology* (EULAR) criteria, as the absence of signs and symptoms attributable to GCA and the normalization of the ESR and CRP values [22]. Relapse was defined as the reappearance of clinical manifestations of GCA that required treatment intensification [23]. Additional outcomes evaluated were the ability to discontinue glucocorticoids and the occurrence of adverse events.

The ESR was considered to be increased when it was higher than 20 or 25 mm/hour for men or women, respectively. A serum CRP value greater than 0.5 mg/dL was considered elevated. Anemia was defined as a hemoglobin level ≤ 11 g/dL, leukopenia as < 4000 leukocytes/µL, lymphopenia as < 1500 lymphocytes/µL, neutropenia as < 1500 neutrophils/µL, and thrombocytopenia as < 100,000 platelets/µL.

### Systematic literature search

A systematic literature search of published randomized controlled trials, non-randomized trials, cohort studies, case series, and case reports was done in MEDLINE/PubMed (https://pubmed.ncbi.nlm.nih.gov/) from the inception of each database to May 31, 2023.

### Statistical analysis

All data were analyzed using descriptive methods. Continuous data were summarized using means, medians, standard deviations (SD), ranges and interquartile ranges (IQR) where appropriate. Categorical data were summarized as numbers and corresponding percentages. Additionally, a comparison between 15 GCA patients from a pilot study by Koster et al. [15] and the 15 GCA patients of our series treated with baricitinib was performed. Continues variables were compared using Mann-Whitney U-test and categorical variables were compared using Fisher´s exact test. Statistical significance was considered as a p-value < 0.05. The analysis was conducted using STATISTICA software (StatSoft Inc. Tulsa, OK, USA).

### Ethical considerations

The study was approved by the Cantabria Clinical Research Ethics Comittee (approval number 2021.414), and was conducted in accordance with the Declaration of Helsinki and the International Conference for Harmonization. All data extracted from the medical records were stored de-identified prior to the analysis. As per the Clinical Research Ethics Committee, this retrospective research did not require informed consent.

## Results

### Baseline general features at JAKi initiation

A total of 35 patients (30 women and 5 men) with GCA who received treatment with JAKi were included. GCA was confirmed by temporal artery biopsy in 15 (62%) patients and by vascular imaging in 24 (69%) patients [Table 1]. Vascular ultrasonography was performed in 15 patients, observing signs of vasculitis in 7 of them. The mean (SD) age at the initiation of JAKi therapy was 72.3 (8.0) years. Overall, 15 (43%) patients received baricitinib (2–4 mg daily), 10 (29%) tofacitinib (5 mg twice a day) and 10 (29%) upadacitinib (15 mg daily). The median (IQR) time from GCA diagnosis to JAKi therapy initiation was 30 (12–48) months. Without considering concomitant glucocorticoid use, JAKi was prescribed as monotherapy in 34 (97%) patients, and combined with methotrexate in one patient. Thus, only one patient who started treatment with baricitinib maintained concurrent treatment with methotrexate at a dose of 10 mg weekly and a prednisone dose of 5 mg/day. Thirty-one patients started treatment with JAKi in combination with glucocorticoids, and three patients initiated JAKi therapy without any other drugs for the treatment of GCA.

**Table 1:** Main features of the 35 GCA patients at JAKi initiation

	Overall *n* = 35	Baricitinib *n* = 15	Tofacitinib *n* = 10	Upadacitinib *n* = 10
Age, years mean ± SD	72.3 ± 8.0	75.6 ± 7.6	67.6 ± 6.3	73.0 ± 8.2
Sex, female/male n (% female)	30/5 (85.7)	14/1 (93.3)	10/0 (100)	6/4 (60)
Time from GCA diagnosis to JAKi initiation (months), median [IQR]	30 [12–48]	32 [12–48]	12 [10.2–45.0]	39 [22.5–75.0]
**CLASSIFICATION/DIAGNOSTIC CRITERIA**				
Criteria used in the GiACTA trial, n (%)	28 (80)	13 (87)	8 (80)	7 (70)
ACR 1990, n (%)	21 (60)	7 (47)	6 (60)	8 (80)
**GCA PHENOTYPE**				
Cranial GCA	15 (43)	6 (17)	4 (40)	5 (50)
Extracranial GCA	7 (20)	3 (9)	2 (20)	2 (20)
Mixed GCA	13 (37)	6 (17)	4 (40)	3 (30)
**POSITIVE TEMPORAL ARTERY BIOPSY, n (%)**	15/24 (62)	6/10 (60)	4/7 (57)	5/7 (71)
**POSITIVE IMAGING TECHNIQUE, n (%)**	24 (69)	11 (73)	6 (60)	7 (70)
Ultrasonography, n positive/n performed (% positive)	7/15 (47)	4/9 (44)	1/2 (50)	2/4 (50)
Positron emission tomography, n positive/n performed (% positive)	16/22 (73)	7/9 (78)	6/7 (86)	3/6 (50)
Magnetic resonance-angiography, n positive/n performed (% positive)	3/9 (33)	1/2 (50)	1/3 (33)	1/4 (25)
Computed tomography-angiography, n positive/n performed (% positive)	4/13 (31)	1/3 (33)	1/2 (50)	2/8 (25)
**CARDIOVASCULAR RISK FACTORS**				
High blood pressure, n (%)	26 (74)	11 (73)	6 (60)	9 (90)
Dyslipidemia, n (%)	16 (46)	9 (60)	2 (20)	5 (50)
Diabetes, n (%)	9 (26)	4 (27)	3 (30)	2 (20)
Previous or current smoking history, n (%)	8 (23)	4 (27)	0 (0)	4 (40)
**SYSTEMIC MANIFESTATIONS**				
PMR, n (%)	12 (34)	4 (27)	4 (40)	4 (40)
Constitutional syndrome, n (%)	10 (29)	7 (47)	2 (20)	1 (10)
Asthenia, n (%)	16 (46)	8 (23)	5 (50)	3 (30)
**CRANIAL AND ISCHEMIC MANIFESTATIONS**				
Headache, n (%)	15 (43)	7 (47)	5 (50)	3 (30)
Jaw claudication, n (%)	6 (17)	1 (7)	2 (20)	3 (30)
Visual symptoms, n (%)	5 (14)	3 (20)	1 (10)	1 (10)
Stroke, n (%)	2 (6)	1 (7)	1 (0)	0 (0)
**LARGE-VESSEL INVOLVEMENT**	20 (57)	10 (67)	6 (60)	4 (40)
**LABORATORY**				
ESR, mm/1st hour, median [IQR]	28 [7–48]	39 [10.5–60.5]	34 [28–48]	7 [2–10]
CRP, mg/dL, median [IQR]	0.9 [0.4–2.5]	1 [0.4–3.5]	0.9 [0.6-2]	0.7 [0.3–1.8]
Hb, g/dL, mean ± SD	12.7 ± 1.7	12.4 ± 1.7	12.5 ± 1.4	13.6 ± 2.0
**Previous synthetic conventional immunosuppressants use, n (%)**				
Methotrexate, n (%)	22 (63)	9 (60)	7 (70)	6 (60)
Leflunomide, n (%)	1 (3)	0 (0)	1 (10)	0 (0)
Antimalarials, n (%)	3 (9)	0 (0)	2 (20)	1 (10)
**Previous biologic immunosuppressants use, n (%)**				
Tocilizumab, n (%)	26 (74)	8 (53)	9 (90)	9 (90)
Sarilumab, n (%)	3 (9)	2 (13)	0 (0)	1 (10)
Adalimumab, n (%)	2 (6)	1 (7)	0 (0)	1 (10)
Abatacept, n (%)	8 (23)	2 (13)	4 (40)	2 (20)
Ustekinumab, n (%)	2 (6)	0 (0)	1 (10)	1 (10)
**GLUCOCORTICOIDS AT JAKi INITIATION**				
Patients on prednisone, n (%)	32 (91)	14 (93)	10 (100)	8 (80)
Prednisone dose, mg/day, median [IQR]	16.2 [8.7–30]	10 [6.2–22.5]	20 [16.2–30]	13.1 [5.6–22.5]

**Abbreviations**: CRP: C-reactive protein; ESR: erythrocyte sedimentation rate; GCA: giant cell arteritis; Hb: hemoglobin; IQR: interquartile range; JAKi: Janus kinase inhibitors; PMR: polymyalgia rheumatica; SD: standard deviation

The main clinical manifestations of the patients at the time of JAKi initiation are summarized in Table 1. Those included headache (*n* = 15 [43%]), jaw claudication (*n* = 6 [17%]), visual symptoms (*n* = 5 [14%]), and PMR symptoms (*n* = 12 [34%]). The median (IQR) baseline serum CRP and ESR values were 0.9 (0.4–2.5) mg/dL and 28 (7–48) mm/hour. The median (IQR) baseline prednisone dose was 16.2 (8.7–30) mg/day.

Before JAKi therapy, 22 (63%) patients had received several conventional synthetic immunosuppressants such as methotrexate (*n* = 22 [63%]), hydroxychloroquine (*n* = 3 [9%]), and leflunomide (*n* = 1 [3%]) [Table 1]. In addition, 30 (86%) patients had been treated with biologics including tocilizumab (*n* = 26 [74%]), sarilumab (*n* = 3 [9%]), abatacept (*n* = 8 [23%]), adalimumab (*n* = 2 [6%]), and ustekinumab (*n* = 2 [6%]) [Table 1].

### Clinical outcomes

**Fig. 1:**
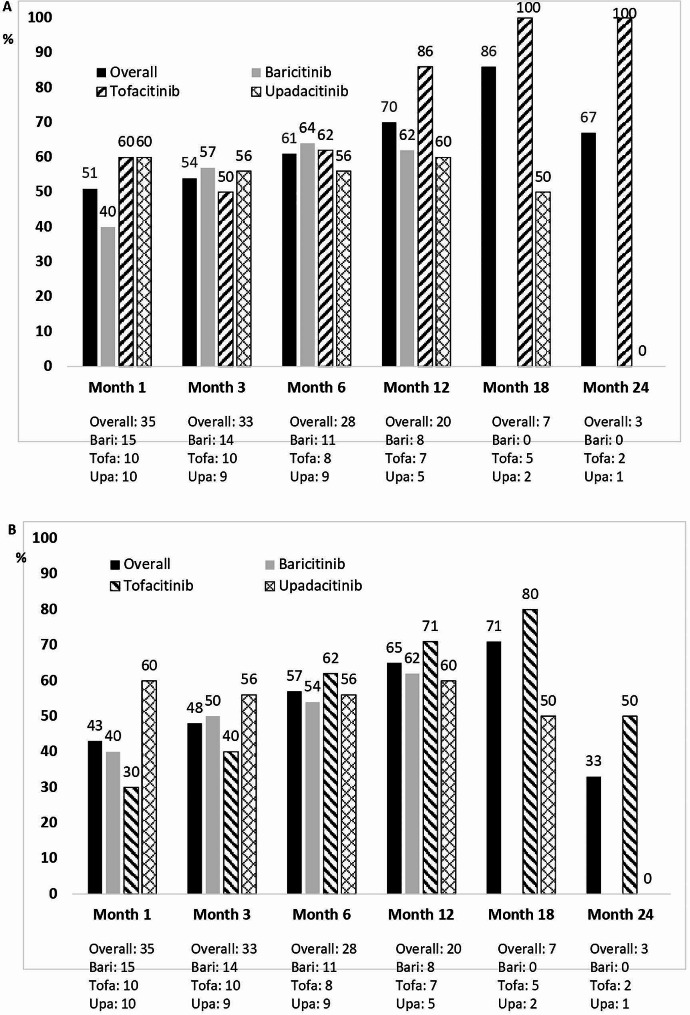
Clinical outcomes of 35 patients with Giant Cell Arteritis after JAK inhibitor initiation. Legend: (**A**) Clinical remission; (**B**) Complete remission. JAK: Janus kinase

Once on JAKi, patients were followed for a median (IQR) period of 11 (6-15.5) months with 35 patients followed for at least one month, 33 patients followed for at least three months, 28 patients followed for at least six months and 20 patients followed for at least twelve months. Most patients experienced improvement of clinical manifestations and laboratory parameters over time following JAKi therapy. Clinical remission was observed at one, three, six and twelve months in 18/35 (51%), 18/33 (54%), 17/28 (61%) and 14/20 (70%) patients, respectively [Fig. 1A]. Complete remission was observed at one, three, six and twelve months in 15/35 (43%), 16/33 (48%), 16/28 (57%) and 13/20 (65%) patients, respectively [Fig. 1B]. Effectiveness was similar across all JAKi [Fig. 1A and B].

**Fig. 2:**
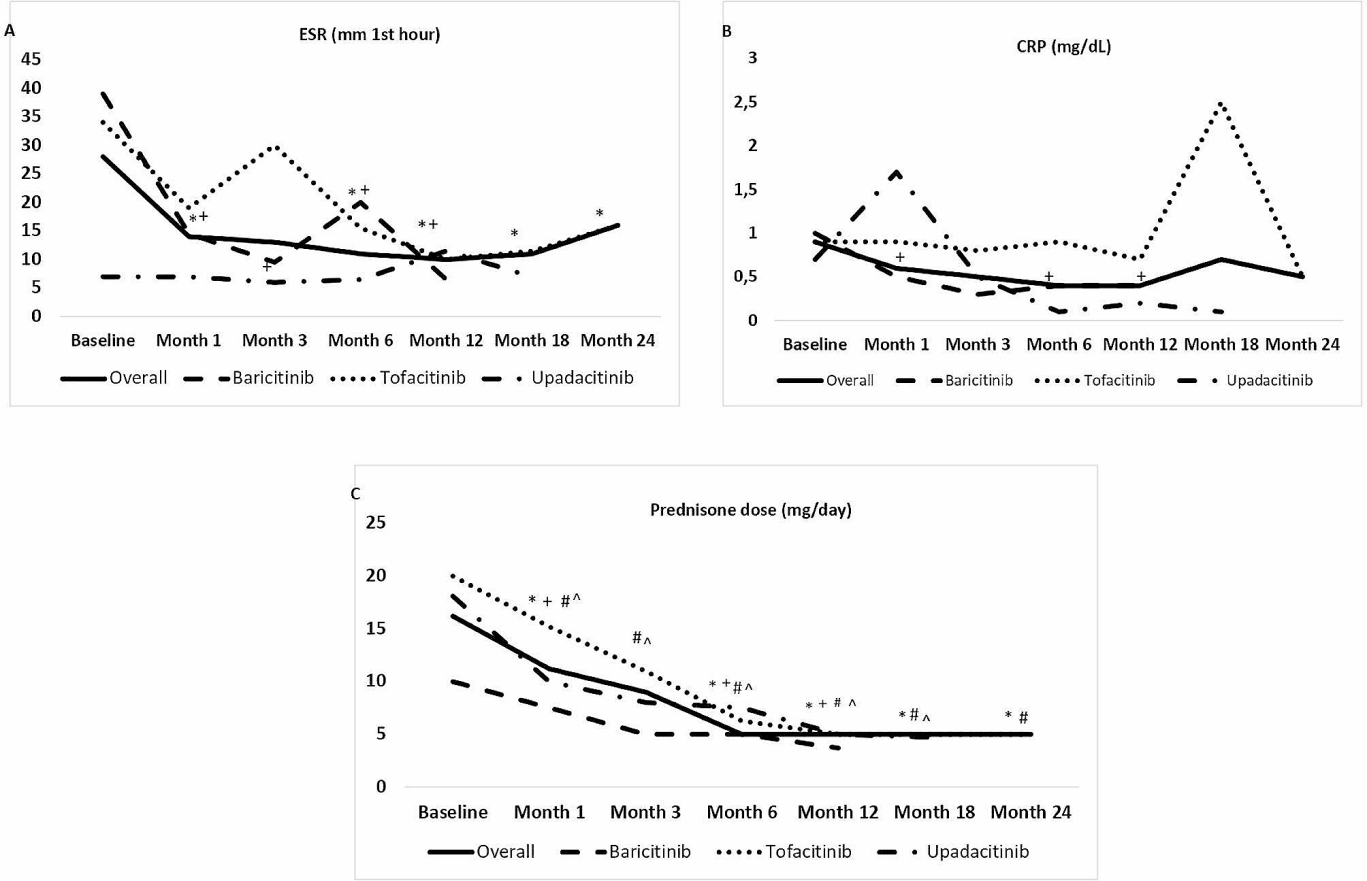
Laboratory abnormalities and reduction of glucocorticoid dose after JAK inhibitor initiation Legend: (data expressed as median values; p compared with baseline). (**A**) Erythrocyte sedimentation rate (ESR); (**B**) Serum C-reactive protein (CRP); and (**C**) Glucocorticoid dose. JAK: Janus kinase. *: *p* < 0.05 in overall series. +: *p* < 0.05 in baricitinib group. #: *p* < 0.05 in tofacitinib group. ^: *p* < 0.05 in upadacitinib group

The median (IQR) ESR declined from 28 (7–48) mm/hour at baseline to 3 (7.2–29.2) mm/hour at last follow-up (*p* < 0.001). The median (IQR) CRP concentration decreased from 0.9 (0.4–2.5) at baseline to 0.4 (0.2–2.1) mg/dL at last follow up (*p* = 0.53) [Fig. 2A and B]. The median (IQR) daily dose of prednisone decreased from 16.2 (8.7–30) at baseline to 5 (0-12.5) mg at last follow up (*p* < 0.001) [Fig. 2C]. In addition, 7 (20%) patients were able to stop glucocorticoids completely.

Overall, eleven (31%) patients discontinued JAKi therapy due to relapse or persistence of active disease. Of these eleven patients, five were on tofacitinib, four on baricitinib, and two on upadacitinib.

### Safety

Adverse events were reported in five (14%) patients during JAKi therapy. One patient on baricitinib developed a urinary tract infection without requiring permanent JAKi discontinuation. The adverse events led to drug discontinuation in the other four patients. These cases included a 74-year-old woman on baricitinib 2 mg/day that developed significant elevation of liver enzymes, a 67-year-old woman on tofacitinib 5 mg twice a day that developed palpitations and dyspnea, a 67-year-old woman on upadacitinib 15 mg daily complicated with disseminated herpes zoster, and a 72-year-old man diagnosed with glioblastoma multiforme six months after starting upadacitinib 15 mg daily. No thromboembolism, major adverse cardiovascular events, or significant cytopenias were observed during follow-up. No cases of GCA-related permanent vision loss were reported either.

### Outcomes in patients previously treated with IL-6R antagonists

Overall, 28 (80%) patients (24 women and 4 men) had previously received treatment with IL-6R blockers including tocilizumab (*n* = 26) and sarilumab (*n* = 3) (one patient received both). Within this group of 28 patients, IL-6R blockade therapy had been stopped due to inefficacy in 20 (71%) of them.

The JAKi prescribed to this subgroup of patients were baricitinib (*n* = 10), tofacitinib (*n* = 9), and upadacitinib (*n* = 9). During a median (IQR) follow-up of 12 (8.7–16.2) months, 16 (57%) out of 28 patients who had previously received IL-6R blockers achieved clinical remission (baricitinib [*n* = 7; 44%], tofacitinib [*n* = 5; 31%], upadacitinib [*n* = 4; 25%]), and 13 (46%) of them complete remission (baricitinib [*n* = 6; 46%], tofacitinib [*n* = 3; 23%], upadacitinib [*n* = 4; 31%]), no observing differences between the three JAKi used. As expected, the ESR (median [IQR] 11 [5–39] mm/hour) and CRP (median [IQR] 0.8 mg/dL [0.3–1.9]) values at the moment of IL-6R blockade therapy discontinuation and initiation of JAKi were normal. Those values further decreased during JAKi therapy (median [IQR] ESR 10 [5.7–22] mm/hour and CRP 0.4 [0.1–1.2] at last follow up, *p* > 0.05 for both comparisons) At the last visit, the median (IQR) dose of prednisone decreased from 15.6 (10–30) mg/day to 5 (0.6–10) mg/day (*p* < 0.001). Of 25 patients who were receiving glucocorticoids at JAKi initiation, six (24%) were able to discontinue them. The median (IQR) daily prednisone dose at last follow up was 5 [0.6–10] mg. JAKi was withdrawn because of GCA relapse in nine (32%) patients. Of these, five patients were receiving tofacitinib, two baricitinib, and 2 upadacitinib.

### Comparison between patients treated with baricitinib in a prospective pilot study and this case series

The main features of the two series of patients are summarized in Supplementary Table 1. The study by Koster et al. was a prospective, proof-of-concept trial of baricitinib (4 mg/day) that enrolled 15 GCA patients with relapsing disease, and employed a relatively rapid (15–22 weeks), structured glucocorticoid taper starting at 10 mg, 20 mg or 30 mg/day [15]. Compared to the patients included in the study by Koster et al., the 15 patients in our series treated with baricitinib had longer disease duration (median [IQR] 32 [12–48] months vs. 9 [7–21] months; *p* = 0.008), less incidence of PMR symptoms at baseline (53% vs. 26%; *p* = 0.010), significantly higher levels of ESR and CRP at baseline (*p* ≤ 0.001), and higher rate of prior immunosuppressive treatment failure at the time of starting baricitinib (*p* < 0.001). As expected given the different study design (prospective clinical trial vs. retrospective real-world study), the prednisone dose at six and twelve months after baricitinib initiation was higher in the patients included in our series. All patients from the study by Koster et al. [15] who completed twelve months of treatment were able to discontinue prednisone, while in our series the median (IQR) daily prednisone dose of the eight patients that received baricitinib for 12 months was 3.7 [0.6–10.6] with only one (14%) patient completely off prednisone by that timepoint (Supplementary Table 1). By week 52, thirteen of the fourteen patients (93%) completing the trial prococol by Koster et al. and five of the eight patients (62%) treated with baricitinib who had reached 52 weeks of in our series were in remission.

## Discussion

The results from this observational study and from the literature review indicate that JAKi may be effective in a sizable proportion of GCA patients, including those that previously failed established glucocorticoid-sparing treatments such as methotrexate and tocilizumab. After a median follow up of nearly one year, approximately 60% of the patients receiving JAKi in our series achieved and maintained clinical remission. In addition, the patients in our cohort were able to significantly reduce their daily prednisone doses to a median of 5 mg and 20% of them weaned off glucocorticoids completely.

Glucocorticoids have been the cornerstone of the treatment of GCA for decades. However, relapses are common when glucocorticoid doses are tapered and adverse events from this type of medications are frequent [24]. In addition, glucocorticoids impair quality of life and are negatively regarded in the long-term by most patients with GCA [24]. Over the last several years, other drugs, such as methotrexate and TNF-$a$ inhibitors have been trialed with controversial or disappointing results [3, 4]. More recently, phase 2 randomized controlled trials with abatacept, mavrilimumab, and secukinumab have shown encouraging preliminary findings that await confirmation [11–13]. The only medication thus far with demonstrated efficacy in a phase 3 randomized controlled trial, however, is tocilizumab [6]. Nevertheless, up to one third of patients relapse while on tocilizumab and up to 10% must discontinue treatment due to adverse events [6, 7, 9, 25]. Furthermore, nearly two thirds of patients experience relapses within 12–24 months after tocilizumab discontinuation [8–10]. The treatment landscape described above underscores the need for additional GCA therapies.

Macrophages and CD4^+^ cell with T helper phenotype type 1 (Th1) and 17 (Th17), which are the main immune cell effectors present in GCA lesions, respond to several cytokines through JAK/STAT pathways, and produce cytokines that in turn amplify the inflammatory response in a JAK/STAT-dependent manner creating a vicious cycle [26, 27]. These cytokines include IL-2, IL-12, IL-23, IL-6, GM-CSF, and IFN-$\gamma$, among others [26, 28–30]. Preclinical investigations have shown downregulation of IFN-$\gamma$, IL-17 and IL-21 leading to decreased CD4^+^ cell infiltrates, neovascularization and intimal proliferation in response to tofacitinib in a model that utilizes a human artery implant on an immunodeficient humanized mouse. Such model recapitulates GCA-like arterial inflammation upon transfusion with peripheral blood mononuclear cells from GCA patients [14].

Clinical data on the efficacy and safety of JAKi in GCA are scarce and limited to a few cases reports, one small retrospective case series, and one small prospective study (Supplementary Table 2) [15–20]. Eriksson et al. [16], evaluated the effectiveness and safety of baricitinib and tofacitinib in 15 relapsing patients with GCA and observed no further relapses, reduction in prednisone use, and improvement in CRP and ESR values during the observation period. Of note, only 20% of these patients had previously received conventional synthetic immunosuppressants and 20% had previously received biologics. Koster et al. [15]. conducted a prospective, open-label, pilot study with baricitinib 4 mg daily for 52 weeks in 15 patients with relapsing GCA. The study employed a pre-specified prednisone taper over 15–22 weeks starting between 10 and 30 mg daily. Fourteen patients completed 52 weeks of treatment and only 1 patient relapsed. The remaining 13 patients achieved and maintained disease remission and were able to discontinue prednisone as per protocol until the end of the study. Noteworthy, only 13% and 7% of the patients enrolled in the trial had previously been treated with conventional synthetic immunosuppressants and biologics, respectively, and the median disease duration of the cohort prior to JAKi therapy was 9 months.

In our series, approximately one third of the patients relapsed while on JAKi and, despite a significant reduction in the daily prednisone dose by the end of follow up, only 20% stopped prednisone completely during the observation period. Possible explanations between our results and the results from the study by Eriksson et al. [16] may include the fact that our cohort of patients could have had more refractory disease reflected by the fact that 63% failed conventional synthetic immunosuppressive agents and 86% failed biological therapy before JAKi initiation. A comparison between our study and the one by Koster et al. [15] is challenging given the markedly different study designs (i.e., retrospective versus prospective), but factors determining what seems to have been an encouraging, yet poorer response to JAKi in our series may also comprise more recalcitrant disease in our cases demonstrated by longer disease duration, and again reflected in the higher exposures to first and second line therapies before JAKi treatment.

Five of the patients treated with JAKi in our series developed adverse events that led to JAKi discontinuation in four of them. Adverse events included two infections (bacterial urinary infection and disseminated varizella-zoster virus infection), one case of liver dysfunction, and one case of glioblastoma multiforme diagnosed after six months of JAKi therapy. No cases of thromboembolism, stroke, myocardial infarction, significant cytopenias, or GCA-related permanent vision loss were observed. Adverse events reported in the study of Eriksson at al [16]. included a case of *Aspergillus fumigatus* infection and a case *Enterococcus faecalis* bacteremia. As expected for a prospective clinical trial, over 90% of patients in the study by Koster et al. [15] reported at least one adverse event during the 52 weeks of follow-up. Those adverse events included infection not requiring antibiotics (*n* = 8), infection requiring antibiotics (*n* = 5), nausea (*n* = 6), leg swelling (*n* = 2), fatigue (*n* = 2), diarrhea (*n* = 1), and abdominal pain (*n* = 1). One patient experienced a severe adverse event consistent of transient thrombocytopenia, which was attributed to concomitant use of antivirals.

The main limitations of our study are its retrospective nature that could have introduced bias due to missing data and the relatively small sample size. In addition, incomplete documentation of data related to individual prednisone tapering courses made the calculation of cumulative prednisone dose, a key outcome measure in GCA, inaccurate and therefore not analyzable. Despite these limitations, information about key efficacy and safety events (e.g., remission, relapse, serious adverse events, and drug discontinuation) were unequivocally present in the data source, which makes our estimations reliable. Moreover, to our knowledge, this is the largest study to date evaluating outcomes of GCA patients treated with JAKi. As such, our results expand prior findings [15, 16], which have been mostly limited to patients naïve to treatment with conventional synthetic immunosuppressants and biologics, suggesting that JAKi can be useful in patients failing those therapies as well.

## Conclusion

In summary, in this retrospective study, JAKi treatment was associated with GCA disease control including reduction in the prednisone use in a sizable proportion of patients, most of whom had failed established glucocorticoid-sparing options including tocilizumab and methotrexate. Until the results of a large phase 3 randomized-controlled trial with upadacitinib (Clinical Trials.gov identifier NCT03725202) become available, our findings may inform clinical decision making for GCA patients in routine practice.

### Electronic supplementary material

Below is the link to the electronic supplementary material.

Supplementary Material 1

## Data Availability

The authors confirm that all data underlying the findings are fully available without restriction. All relevant data are included in the paper.

## Abbreviations

CRP: C-reactive protein
CTA: Computed tomography angiography
ESR: Erythrocyte sedimentation rate
EULAR: European League Against Rheumatism
FDG: Fluorodeoxyglucose
GCA: Giant cell arteritis
GM-CSF: Granulocyte-macrophage colony-stimulating factor
IL-6R: IL-6 receptor
IFN: Interferon
IQR: Interquartile range
JAKi: JAK inhibitors
JAK/STAT: Janus kinase/signal transducers and activators of transcription
MRI/MRA: Magnetic resonance imaging/angiography
PET: Positron emission tomography
PMR: Polymyalgia rheumatica
SD: Standard deviation
Th1: T helper type 1
Th17: T helper type 17
TNF: tumor necrosis factor
