# Supplementary material for: Effectiveness of janus kinase inhibitors in relapsing giant cell arteritis in real-world clinical practice and review of the literature
Source: Arthritis Res Ther. 2024 Jun 5;26:116. doi: 10.1186/s13075-024-03314-9 (PMC11151571; doi:10.1186/s13075-024-03314-9)
Supplement: Supplementary file 1 — Supplementary Material 1 [file 13075_2024_3314_MOESM1_ESM.docx]

**SUPPLEMENTARY MATERIAL**

**Suplemmentary Table 1.** Comparison between the GCA patients included in prospective baricitinib study and the 15 GCA patients of our series treated with baricitinib.

|  | **Koster et al***  **n=15** | **Current series**  **n=15** | **p** |
| --- | --- | --- | --- |
| Age, years mean ± SD | 72·4±7·2 | 75·6±7·6 | 0·12 |
| Sex, female/male n (% female) | 11/4 (73) | 14/1 (93) | 0·17 |
| Time from GCA diagnosis to JAKi initiation (months), median [IQR] | 9 [7-21] | 32 [12-48] | 0·008 |
| AT THE MOMENT OF JAKi INITIATION |  |  |  |
| Systemic manifestations |  |  |  |
| PMR, n (%) | 8 (53) | 4 (26) | 0·010 |
| Constitutional symptoms, n (%) | 8 (53) | 6 (40) | 0·36 |
| Cranial manifestations |  |  |  |
| Headache, n (%) | 6 (40) | 6 (40) | 0·99 |
| Jaw claudication, n (%) | 1 (7) | 1 (7) | 0·99 |
| Visual symptoms, n (%) | ND | 3 (20) | - |
| Positive temporal artery biopsy, n (%) | 10/14 (71) | 6/10 (60) | 0·42 |
| Large-vessel imaging suggesting vasculitis, n (%) | 10/15 (67) | 9/10 (90) | 0·12 |
| Laboratory data |  |  |  |
| ESR (mm 1st hour), median [IQR)] | 7 [6-17] | 39 [10·5-60·5] | <0·001 |
| CRP (mg/dL), median [IQR] | 0·3 [0·3-0·7] | 1 [0·4-3·0] | 0·001 |
| Glucocorticoids at JAKi initiation |  |  |  |
| Patients on prednisone, n (%) | 15 (100) | 14 (93) | 0·80 |
| Prednisone dose, mg/day, median [IQR] | 20 [10-30] | 10 [6·2-22·5] | 0·24 |
| Previous conventional synthetic immunosuppressants use, n (%) | 2 (13) | 9 (60) | <0·001 |
| Previous biologic immunosuppressants use, n (%) | 1 (7) | 11 (73) | <0·001 |
| FOLLOWING JAKi INITIATION |  |  |  |
| Prednisone dose (mg/day) at month 6, median [IQR] | 0 [0-0] | 5 [1·9-8·7] | <0·001 |
| Prednisone dose (mg/day) at month 12, median [IQR] | 0 [0-0] | 3·7 [0·6-10·6] | 0·007 |
| ESR (mm 1st hour) at month 6, median [IQR] | 13 [7-19] | 20 [9·5-28·5] | 0·13 |
| ESR (mm 1st hour) at month 12, median [IQR] | 10 [5-17] | 6·5 [4·7-10·2] | 0·26 |
| CRP (mg/dL) at month 6, median [IQR] | 0·3 [0·3-0·3] | 0·4 [0·3-0·6] | 0·053 |
| CRP (mg/dL) at month 12, median [IQR] | 0·3 [0·3-0·3] | 0·4 [0·2-0·6] | 0·033 |
| Discontinued prednisone at month 6, n/N available (%) | 14/14 (100) | 1/10 (10) | <0·001 |
| Discontinued prednisone at month 12, n/N available (%) | 14/14 (100) | 1/7 (14) | <0·001 |
| Clinical remission at month 12, n/N (%) | 13/14 (93) | 5/8 (63) | 0·117 |

**Abbreviations:** CRP: C-reactive protein; ESR: erythrocyte sedimentation rate; IQR: interquartile range; JAKi: Janus kinase inhibitors; PMR: polymyalgia rheumatica; SD: standard deviation. *Reference 15.

**Supplemantary Table 2.** Current series and literature review of patients with GCA treated with JAKi.

| **Reference** | **Number of cases** | **Sex** | **Age,**  **mean±SD** | **JAKi received** | **Previous conventional synthetic immunosuppressive drugs** | **Previous biologic drugs** | **Follow-up (months), mean±SD** | **Outcome** |
| --- | --- | --- | --- | --- | --- | --- | --- | --- |
| Herlihy et al (18) | 1 | Female | 75 | Ruxolitinib | Methothrexate, mycophenolate mophetil | None | 9 | No outcome data reported |
| Prigent et al (17) | 1 | Female | 76 | Baricitinib | Methothrexate | Tocilizumab | 12 | Clinical improvement |
| Camellino et al (19) | 3 | Female (n=3) | 74±11·5 | Baricitinib (n=3) | Methothrexate (n=2), hydroxychloroquine (n=1), sulfasalazine (n=1), cyclosporine (n=1), mycophenolate mophetil (n=1) | Tocilizumab (n=2) | 8·5±4·9; (no data in 1 patient) | Clinical improvement (n=1); no outcome data reported (n=2) |
| Koster et al (15) | 15 | Female (n=11), male (n=4) | 72·4±7·2 | Baricitinib (n=15) | Methothrexate (n=2), Cyclophosphamide (n=1) | Sirukumab (n=1) | 11·3±2·3 | Clinical improvement (n=13); no improvement (n=1); no outcome data reported (n=1) |
| Eriksson et al (16) | 15 | Female (n=8), male (n=3) | 70·2±6·0 | Baricitinib / tofacitinib | Methotrexate (n=3) | Tocilizumab (n=3), infliximab (n=1) | 19±10·5 | Clinical improvement (n=15) |
| Sanada et al (20) | 1 | Female | 72 | Upadacitinib | Sulfasalazine | None | 7·5 | Clinical improvement |
| Current series | 35 | Female (n=30), male (n=5) | 72·3±8·0 | Baricitinib (n=15), tofacitinib (n=10), upadacitinib (n=10) | Methothrexate (n=22), hydroxychloroquine (n=3),  leflunomide (n=1) | Tocilizumab (n=26), sarilumab (n=3), abatacept (n=8), adalimumab (n=2), ustekinumab (n=2) | 11·4±6·8 | Clinical improvement (n=22); no improvement (n=11) |
